# Interstitial Fibrosis Severity Is Not Independently Associated with Anemia in Biopsy-Proven Primary Glomerulonephritis: A Nationwide Registry Analysis

**DOI:** 10.3390/medicina62050820

**Published:** 2026-04-25

**Authors:** Egemen Cebeci, Kenan Turgutalp, Savaş Öztürk, Yasemin Özlük, Sibel Gökçay Bek, Abdullah Şumnu, Nurhan Seyahi, Mahmut Yavuz, Serhan Pişkinpaşa, Ömer Faruk Akçay, Tamer Sakacı, Garip Şahin, Bülent Tokgöz, Gülizar Şahin, İlter Bozacı, Belda Dursun, Savaş Sipahi, Arzu Özdemir, Gültekin Süleymanlar, Sena Ulu, Fatma Betül Güzel, Sim Kutlay, Ergün Parmaksız, İlhan Kurultak, Nedim Yılmaz Selçuk, Yaşar Yıldırım, Meltem Gürsu, Caner Çavdar, Meryem Timuçin, Zeki Aydın, Deren Oygar, Serdar Kahvecioğlu, Müge Üzerk Kibar, Dilek Torun, Dilek Taymez, Mehmet Küçük, Serap Demir, Leyla Koç, Siren Sezer, Murat Duranay, Simge Bardak, Lütfullah Altıntepe, Mehmet Koç, Alper Azak, Ali Rıza Odabaş, Zülfikar Yılmaz, Saime Paydaş

**Affiliations:** 1 Division of Nephrology, Istanbul Haseki Training and Research Hospital, University of Health Sciences, 34260 Istanbul, Türkiye; 2 Division of Nephrology, Mersin University Faculty of Medicine, 33343 Mersin, Türkiye; k.turgutalp@hotmail.com (K.T.);; 3 Division of Nephrology, Istanbul University Istanbul Faculty of Medicine, 34093 Istanbul, Türkiye; savasozturkdr@yahoo.com; 4 Division of Pathology, Istanbul University Istanbul Faculty of Medicine, 34093 Istanbul, Türkiye; yasozluk@gmail.com; 5 Division of Nephrology, Kocaeli University Faculty of Medicine, 41380 Kocaeli, Türkiye; 6 Division of Nephrology, Faculty of Medicine, Medipol University, 34810 İstanbul, Türkiye; asumnu@medipol.edu.tr; 7 Division of Nephrology, Cerrahpaşa Faculty of Medicine, Istanbul Cerrahpaşa University, 34098 İstanbul, Türkiye; nseyahi@yahoo.com; 8 Division of Nephrology, Bursa Uludag University Faculty of Medicine, 16059 Bursa, Türkiye; myavuz@uludag.edu.tr; 9 Division of Nephrology, Ankara Numune Training and Research Hospital, 06100 Ankara, Türkiye; svppasa@yahoo.com; 10 Division of Nephrology, Gazi University Faculty of Medicine, 06500 Ankara, Türkiye; 11 Division of Nephrology, Hamidiye Etfal Training and Research Hospital, University of Health Sciences, 34303 Istanbul, Türkiye; sakacitamer@hotmail.com; 12 Division of Nephrology, Faculty of Medicine, Eskişehir Osmangazi University, 26480 Eskişehir, Türkiye; gsahin@ogu.edu.tr; 13 Division of Nephrology, Faculty of Medicine, Erciyes University, 38039 Kayseri, Türkiye; 14 Division of Nephrology, Sultan 2. Abdulhamid Han Training and Research Hospital, University of Health Sciences, 34668 Istanbul, Türkiye; 15 Division of Nephrology, Bozyaka Training and Research Hospital, University of Health Sciences, 35170 Izmir, Türkiye; ilterbozaci@gmail.com; 16 Division of Nephrology, Faculty of Medicine, Pamukkale University, 20160 Denizli, Türkiye; belda1@yahoo.com; 17 Division of Nephrology, Faculty of Medicine, Sakarya University, 54290 Sakarya, Türkiye; savassipahi@yahoo.com; 18 Division of Nephrology, Bakırköy Dr. Sadi Konuk Training and Research Hospital, 34147 Istanbul, Türkiye; 19 Division of Nephrology, Faculty of Medicine, Akdeniz University, 07070 Antalya, Türkiye; gsuleymanlar@akdeniz.edu.tr; 20 Division of Nephrology, Faculty of Medicine, Afyon University, 03200 Afyon, Türkiye; drsenaulu@yahoo.com; 21 Division of Nephrology, Faculty of Medicine, Kahramanmaras Sutcu Imam University, 46100 Kahramanmaras, Türkiye; fatmabetulduygu@hotmail.com; 22 Division of Nephrology, İbni Sina Hospital, Ankara University, 06100 Ankara, Türkiye; skutlay@hotmail.com; 23 Division of Nephrology, Kartal Lütfi Kırdar Training and Research Hospital, 34865 İstanbul, Türkiye; drergnprmksz@hotmail.com; 24 Division of Nephrology, Faculty of Medicine, Trakya University, 22030 Edirne, Türkiye; ilhankurultak@trakya.edu.tr; 25 Division of Nephrology, Faculty of Medicine, Necmettin Erbakan University, 42080 Konya, Türkiye; 26 Division of Nephrology, Faculty of Medicine, Dicle University, 21280 Diyarbakır, Türkiyedrzulf21@gmail.com (Z.Y.); 27 Division of Nephrology, Faculty of Medicine, Bezmialem Vakif University, 34093 Istanbul, Türkiye; meltem1401@yahoo.com; 28 Division of Nephrology, Faculty of Medicine, Dokuz Eylül University, 35340 İzmir, Türkiye; caner.cavdar@deu.edu.tr; 29 Division of Nephrology, Faculty of Medicine, Cumhuriyet University, 58140 Sivas, Türkiye; 30 Division of Nephrology, Darica Farabi Training and Research Hospital, University of Health Sciences, 41700 Kocaeli, Türkiye; zekiaydindr@yahoo.com; 31 Division of Nephrology, Dr Burhan Nalbantoglu State Hospital, 99010 Lefkosa, Cyprus; derenoygar@yahoo.com; 32 Division of Nephrology, Bursa Yüksek İhtisas Training and Research Hospital, 16110 Bursa, Türkiye; serdar.kahvecioglu@sbu.edu.tr; 33 Division of Nephrology, Faculty of Medicine, Hacettepe University, 06100 Ankara, Türkiye; 34 Division of Nephrology, Başkent University, Dr. Turgut Noyan Hospital, 01250 Adana, Türkiye; dilektorun@hotmail.com; 35 Division of Nephrology, Kocaeli State Hospital, 41040 Kocaeli, Türkiye; guvendilek@yahoo.com; 36 Division of Nephrology, Okmeydanı Training and Research Hospital, 34384 İstanbul, Türkiye; mehmet.kucuk@sbu.edu.tr; 37 Division of Nephrology, Gaziosmanpaşa Training and Research Hospital, 34437 İstanbul, Türkiye; leylaakdenizdr1@gmail.com; 38 Division of Nephrology, Faculty of Medicine, Başkent University, 06490 Ankara, Türkiye; sirensezer@outlook.com; 39 Division of Nephrology, Ankara Training and Research Hospital, University of Health Sciences, 06230 Ankara, Türkiye; duranaymurat@hotmail.com; 40 Division of Nephrology, Batman State Hospital, 72070 Batman, Türkiye; bardaksimge@gmail.com; 41 Division of Nephrology, Faculty of Medicine, Selçuk University, 42130 Konya, Türkiye; laltintepe@yahoo.com; 42 Division of Nephrology, Faculty of Medicine, Marmara University, 34854 Istanbul, Türkiye; 43 Division of Nephrology, Balıkesir Training and Research Hospital, 10145 Balıkesir, Türkiye; dralperazak@gmail.com; 44 Division of Nephrology, İstanbul Medeniyet University, 34722 İstanbul, Türkiye; aliriza.odabas@medeniyet.edu.tr; 45 Division of Nephrology, Faculty of Medicine, Çukurova University, 01330 Adana, Türkiye; spaydas@cu.edu.tr

**Keywords:** anemia, glomerulonephritis, hemoglobin, interstitial fibrosis

## Abstract

*Background and Objectives*: Anemia is a frequent complication of chronic kidney disease (CKD), primarily attributed to erythropoietin deficiency. Interstitial fibrosis (IF), which disrupts the renal interstitium where erythropoietin-producing cells reside, may contribute to anemia independent of glomerular filtration rate (GFR). However, data in primary glomerulonephritis (PGN) are limited and conflicting. *Materials and Methods*: In this nationwide multicenter registry analysis (TSN-GOLD), 2794 adults with biopsy-proven PGN were included. Interstitial fibrosis was graded semi quantitatively (0–3). Anemia was defined according to KDIGO/WHO criteria. Multivariable logistic regression models were constructed to evaluate the independent association between IF severity and anemia, adjusting for age, sex, eGFR, log-transformed proteinuria, hypertension, diabetes mellitus, and biopsy diagnosis. Interaction between IF and eGFR was assessed. A predefined subgroup analysis was performed in patients with preserved renal function (eGFR ≥ 60 mL/min/1.73 m^2^). *Results*: Anemia was present in 34.4% of patients. Although moderate-to-severe IF was more frequent among anemic patients (*p* < 0.001), IF severity was not independently associated with anemia in multivariable analysis (*p*-trend = 0.72). Female sex and lower eGFR were independently associated with anemia. A statistically significant IF×eGFR interaction was observed (*p* = 0.0029), indicating effect modification across renal function levels. The model demonstrated moderate discrimination (AUC = 0.705). In patients with preserved renal function, IF severity was not associated with anemia. *Conclusions*: In this large multicenter cohort of PGN patients, interstitial fibrosis severity was not independently associated with anemia after adjustment for renal function and clinical covariates. These findings suggest that the association between interstitial fibrosis and anemia in PGN appears largely mediated by renal functional status rather than fibrosis severity alone.

## 1. Introduction

Anemia is one of the most common complications of chronic kidney disease (CKD), second only to hypertension, and the stage of CKD is the strongest determinant of its prevalence. Previous studies have reported that 21–62% of non-dialysis-dependent CKD patients have anemia, with prevalence increasing progressively as kidney function declines [1,2,3]. In addition to CKD stage, female sex, black race, and advanced age (≥75 years) are independently associated with a higher prevalence of anemia [4,5]. Beyond disease stage, the underlying etiology of CKD also influences anemia risk. Anemia is more frequently observed in patients with diabetic nephropathy, whereas it appears to be less common in hypertensive nephropathy and autosomal dominant polycystic kidney disease. In a study including 2420 non-dialysis-dependent CKD patients, the prevalence of anemia among those with stage 3 CKD was 58.7% in diabetic nephropathy, compared with 36.6% in hypertensive nephropathy and 51.9% in glomerulonephritis [6].

The principal mechanisms underlying anemia in CKD include relative erythropoietin (EPO) deficiency, reduced responsiveness to EPO, and iron deficiency. Chronic inflammation and uremic toxins may further impair cellular responsiveness to EPO, contributing to functional EPO resistance. EPO is synthesized by peritubular interstitial cells located in the renal cortex, which have been identified as peritubular fibroblasts [7,8,9]. These fibroblasts are interstitial mesenchymal cells responsible for producing extracellular matrix and structurally supporting the epithelium. Experimental models of interstitial nephropathy have demonstrated that interstitial fibroblasts exhibit reduced capacity for EPO production following renal injury [10]. Therefore, structural alterations within the renal interstitium―particularly interstitial fibrosis―may directly compromise EPO synthesis.

Notably, in diabetic CKD, anemia tends to occur more frequently and at earlier stages compared with other etiologies [11,12]. Although the precise mechanisms remain incompletely understood, proposed contributors include EPO deficiency, iron deficiency, EPO resistance, inhibition of hypoxia-inducible factor signaling due to hyperglycemia, and tubulointerstitial injury mediated by advanced glycation end-products [13,14]. Inomata et al. [15] reported that interstitial fibrosis contributes to anemia in diabetic nephropathy. Similarly, Mise et al. [16] demonstrated, in biopsy-proven diabetic nephropathy, that higher interstitial fibrosis and tubular atrophy (IFTA) scores were more strongly associated with lower hemoglobin levels in multivariable models adjusted for clinical and histopathological variables. Furthermore, in a retrospective analysis of patients with biopsy-proven diabetic nephropathy, lower hemoglobin levels were associated with an increased risk of renal events and mortality in those with severe IFTA compared to those with mild IFTA [17].

In contrast, data regarding the relationship between tubulointerstitial injury and anemia in patients with primary glomerulonephritis (PGN) are limited. In a cohort of 462 patients with IgA nephropathy, female sex, hypoalbuminemia, reduced estimated glomerular filtration rate (eGFR), and severe renal tubulointerstitial lesions were independently associated with renal anemia; notably, tubulointerstitial involvement exceeding 50% was associated with a 2.57-fold increased risk of anemia [18]. In a single-center biopsy-based study conducted in our country, interstitial fibrosis (IF) severity correlated with lower hemoglobin levels in PGN; however, IF was not identified as an independent determinant in multivariable analyses [19]. Thus, existing evidence in PGN remains limited and partially conflicting, and the independent impact of interstitial fibrosis severity on anemia across different histopathological grades has not been fully elucidated.

In patients with primary glomerulonephritis, fibrosis of the renal interstitium may contribute to anemia development; however, whether this association is independent of renal function decline remains uncertain. Accordingly, in this multicenter registry analysis, we aimed to evaluate the association between interstitial fibrosis severity and anemia parameters in patients with primary glomerulonephritis, independent of renal function indices.

## 2. Materials and Methods

### 2.1. Study Population

For the purposes of this nationwide retrospective multicenter study, data were obtained from the registry of the Glomerular Diseases Working Group of the Turkish Society of Nephrology (TSN-GOLD). This study follows the Strengthening the Reporting of Observational Studies in Epidemiology (STROBE) reporting guideline. The TSN-GOLD registry and the studies derived from its data were approved by Istanbul University Istanbul Faculty of Medicine Ethical Committee (2011/1164), and complied with the Declaration of Helsinki and its later amendments. A total of 4399 patients from 47 centers recorded in the TSN-GOLD registry between May 2009 and May 2019 were initially evaluated. After exclusion of 524 patients without complete light microscopy and immunofluorescence findings, 3875 patients with biopsy-proven primary glomerulonephritis remained eligible. Patients with minimal change disease (*n* = 259), acute proliferative glomerulonephritis (*n* = 31), and crescentic glomerulonephritis (*n* = 244) were excluded due to distinct clinicopathological characteristics. An additional 547 patients were excluded because of missing interstitial fibrosis grading and/or hemoglobin data. Consequently, 2794 patients from 45 centers with complete clinical, laboratory, and histopathological data were included in the final analysis.

### 2.2. Clinical Data Collection

Demographic, clinical and laboratory characteristics of all patients were obtained and entered into the registry by an attending nephrologist at every center. Sex, age at biopsy, height (cm), weight (kg), and body mass index (BMI; calculated as weight [kg]/height [m]^2^) were recorded. Comorbid conditions, including hypertension and diabetes mellitus, as well as the presence of edema at the time of biopsy, were documented. Use of renin–angiotensin system inhibitors, including angiotensin-converting enzyme inhibitors and angiotensin receptor blockers, at the time of biopsy was recorded. Laboratory parameters at biopsy included blood urea nitrogen, serum creatinine, eGFR, uric acid, albumin, alanine aminotransferase, LDL cholesterol, HDL cholesterol, hemoglobin, hematocrit, and proteinuria. eGFRs were calculated by using the Chronic Kidney Disease Epidemiology Collaboration (CKD-EPI) 2009 formula [20]. Proteinuria was analyzed as the recorded daily proteinuria value (mg/day) at the time of biopsy. At examination of urinary sediment, microscopic hematuria was described as >5 erythrocytes per high power field and leukocyturia was described as >5 leukocytes per high power field. Anemia was defined according to KDIGO anemia guideline / World Health Organization criteria as a hemoglobin level <13 g/dL in men and <12 g/dL in women [21,22].

### 2.3. Histopathological Evaluation

A nephropathologist at every center evaluated individual kidney biopsies. Histopathological details were gathered from the individual biopsy data. IF and tubular atrophy (TA) were graded using a semiquantitative scale from 0 to 3: 0, normal; 1 (mild), <25% of interstitium; 2 (moderate), 25–50%; and 3 (severe), >50%. Histopathological variables included total number of glomeruli, globally sclerotic glomeruli, and segmentally sclerotic glomeruli.

### 2.4. Statistical Analysis

Continuous variables were evaluated for normality using visual inspection of histograms and the Shapiro–Wilk test. As most variables were not normally distributed, continuous data are presented as median (interquartile range, IQR; 25th–75th percentile). Between-group comparisons for continuous variables were performed using the Mann–Whitney U test. Categorical variables are presented as counts and percentages and were compared using the chi-square test or Fisher’s exact test, as appropriate.

Multivariable logistic regression analysis was performed to identify independent predictors of anemia. Covariates were selected a priori based on clinical relevance and prior literature and included age, sex, estimated glomerular filtration rate (eGFR), log-transformed proteinuria [ln(mg/day + 1)], hypertension, diabetes mellitus, biopsy diagnosis, and interstitial fibrosis (IF) severity (normal, mild, moderate, severe). Continuous predictors were modeled as linear terms on the log-odds scale, and the linearity assumption was assessed visually.

Missing data were minimal for most covariates (<5%), except for proteinuria (8.2%). Given the relatively low overall level of missingness and the large sample size, multivariable analyses were performed using complete-case data. Multicollinearity was assessed using variance inflation factors (VIF), with all VIF values < 2 indicating no evidence of significant multicollinearity. Model discrimination was evaluated using the area under the receiver operating characteristic curve (AUC).

To explore potential effect modification, an interaction term between IF severity and eGFR was introduced into the regression model. To reduce collinearity between the interaction components, eGFR was mean-centered prior to inclusion in the interaction model.

A predefined subgroup analysis was conducted in patients with preserved renal function (eGFR ≥ 60 mL/min/1.73 m^2^). Because eGFR was used to define the subgroup (range restriction), it was excluded from the subgroup regression model to avoid overadjustment and collinearity.

Adjusted predicted probabilities of anemia were estimated from the final interaction model and plotted across the observed eGFR range for IF grades 0–3 while holding other covariates constant at their mean (continuous variables) or reference (categorical variables) values.

All analyses were performed using SPSS for Windows (version 25.0; IBM Corp., Armonk, NY, USA) and Python (version 3.9; Python Software Foundation, Wilmington, DE, USA). All statistical tests were two-sided, and a *p*-value < 0.05 was considered statistically significant.

## 3. Results

### 3.1. Study Population

A total of 2794 patients with biopsy-proven primary glomerulonephritis were included. The median age was 40.0 years (IQR 29.9–51.5), and 56.9% were male. Median serum creatinine was 1.0 mg/dL (IQR 0.7–1.5), corresponding to a median eGFR of 85.6 mL/min/1.73 m^2^ (IQR 52.1–113.1). Median proteinuria was 3423 mg/day (IQR 1544–6400). Median hemoglobin was 13.1 g/dL (IQR 11.9–14.5).

### 3.2. Baseline Characteristics According to Anemia Status

Of the 2794 patients, 960 (34.4%) were classified as anemic and 1834 (65.6%) as non-anemic. Median hemoglobin levels were significantly lower in the anemic group compared with the non-anemic group (11.2 g/dL [IQR 10.3–11.9] vs. 14.0 g/dL [IQR 13.1–15.0], *p* < 0.001) and median hematocrit levels were significantly lower in the anemic group compared with the non-anemic group (33% [IQR 31–36] vs. 41% [IQR 39–44]), *p* < 0.001). Anemic patients were older and more frequently female. Hypertension and edema were more common among anemic patients. Serum creatinine was higher and eGFR was lower in the anemic group (median eGFR 60.5 vs. 94.8 mL/min/1.73 m^2^; *p* < 0.001). The prevalence of anemia differed across CKD stages, with proportions of 23.4%, 28.5%, 47.4%, 72.2%, and 86.8% in stages 1 through 5, respectively. (*p* < 0.001). Baseline characteristics are presented in Table 1.

### 3.3. Relationship Between Interstitial Fibrosis and Anemia Status

**Figure 1:**
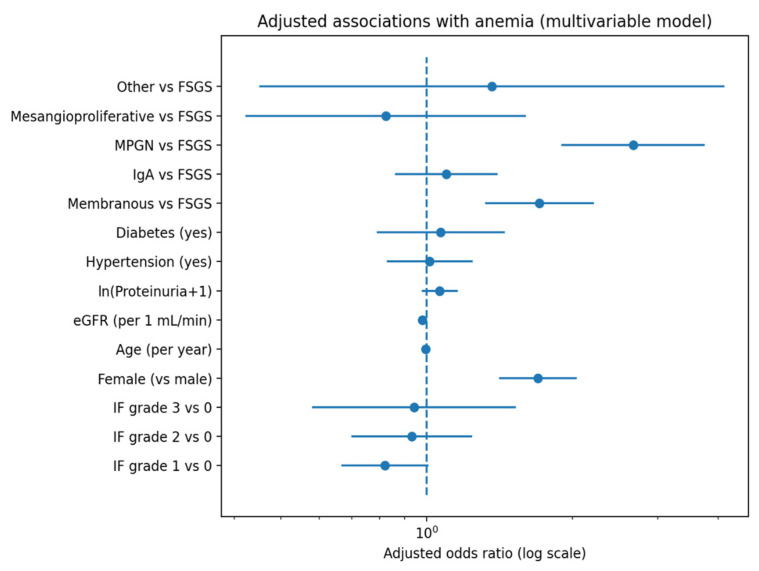
Forest plot of adjusted odds ratios for anemia from the multivariable logistic regression model. Adjusted odds ratios (ORs) with 95% confidence intervals for anemia from the main multivariable logistic regression model. The vertical dashed line indicates OR = 1. IF grades are shown relative to IF grade 0, and biopsy diagnosis categories are shown relative to FSGS (reference). ORs are displayed on a logarithmic scale.

Regarding histopathology, moderate-to-severe IF and TA were more frequent among anemic patients (*p* < 0.001 for both distributions). In the complete-case multivariable model including 2486 patients (AUC = 0.705), interstitial fibrosis severity was not independently associated with anemia (Table 2). Forest plot of adjusted odds ratios for anemia from the multivariable logistic regression model are presented in Figure 1. Modeling IF severity as an ordinal variable likewise demonstrated no significant linear trend between increasing fibrosis grade and anemia risk (β (log-odds) = 0.018; OR per grade = 1.02 (95% CI 0.91–1.14), *p*-trend = 0.72).

Female sex (OR 1.66, 95% CI 1.38–2.00; *p* < 0.001), lower eGFR (OR 0.98 per 1 mL/min increase; *p* < 0.001) were independently associated with anemia.

In the alternative model including IF as a binary variable, IF remained non-significant (OR 0.94, 95% CI 0.77–1.14; *p* = 0.52; AUC = 0.704) (Table 3).

### 3.4. Interaction and Subgroup Analyses

**Figure 2:**
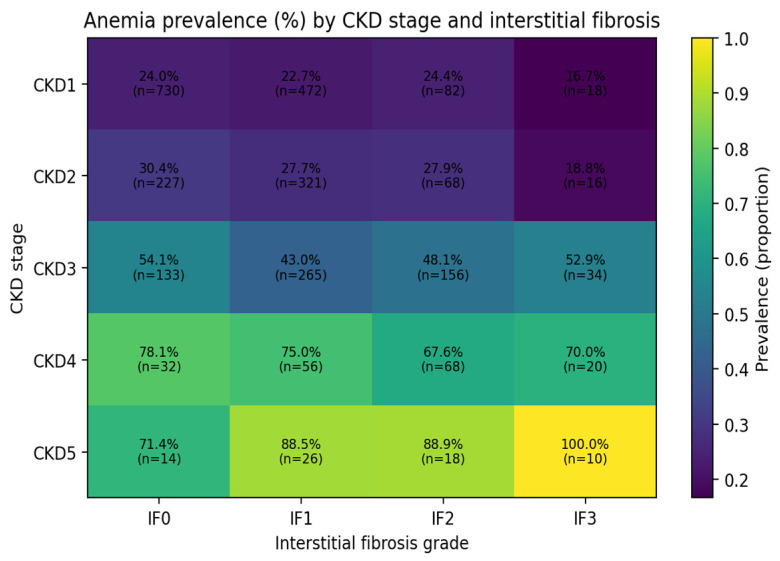
Heatmap of anemia prevalence by CKD stage and interstitial fibrosis grade. Heatmap showing the observed anemia prevalence (color intensity) across the cross-classification of CKD stage (rows) and IF grade (columns). Each cell is annotated with prevalence (%) and the number of patients (*n*) contributing to that estimate. This figure illustrates effect heterogeneity of anemia prevalence across combined structural (IF) and functional (CKD stage) strata.

Lower eGFR was independently associated with anemia (OR 0.979 per 1 mL/min increase, *p* < 0.001) (Table 2). Expressed in clinically meaningful terms, each 10 mL/min/1.73 m^2^ decrease in eGFR corresponded to approximately 20% higher odds of anemia. Anemia prevalence across the cross-classification of CKD stage and IF grade are presented in Figure 2.

**Figure 3:**
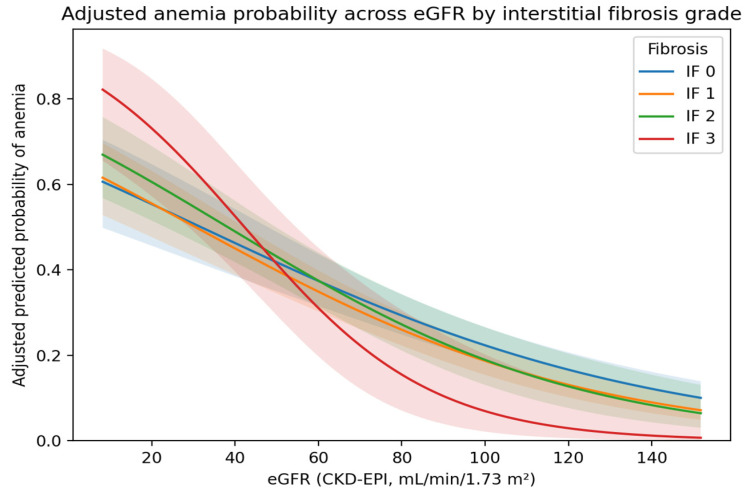
Adjusted predicted probability of anemia across eGFR by interstitial fibrosis grade (IF×eGFR interaction model). Model-based adjusted predicted probability of anemia across the eGFR spectrum (CKD-EPI, mL/min/1.73 m^2^), stratified by IF grades 0–3. Predictions were derived from a multivariable logistic regression model including an IF×eGFR interaction term; other covariates were held constant at their mean values (continuous) or reference categories (categorical). Shaded bands denote 95% confidence intervals.

A statistically significant interaction between IF severity and eGFR was observed (β = −0.005; OR 0.995 per 1 mL/min increase; 95% CI 0.992–0.998; *p* = 0.0029). Adjusted predicted probabilities derived from the interaction model are presented in Figure 3.

Among 1722 patients (subgroup) with preserved renal function (eGFR ≥ 60 mL/min/1.73 m^2^) included in the adjusted model (AUC = 0.604), IF severity was not associated with anemia (Table 4).

## 4. Discussion

In this nationwide cohort of patients with biopsy-proven primary PGN, IF severity was more frequently observed among anemic patients in unadjusted analyses; however, IF severity was not independently associated with anemia after multivariable adjustment. These findings indicate that the crude association between IF and anemia in PGN is largely explained by coexisting clinical and functional determinants―particularly renal function―rather than by fibrosis grade per se. While this observation differs from biopsy-based studies in diabetic nephropathy, in which advanced tubulointerstitial lesions have been strongly linked to anemia and lower hemoglobin levels in adjusted models [15,16,17], it is consistent with prior evidence in PGN populations demonstrating that IF correlates with hemoglobin levels in univariate analyses but does not remain an independent predictor after adjustment for confounding factors [19]. Importantly, data examining the relationship between tubulointerstitial injury and anemia in PGN are limited and partially conflicting. Although a significant association between severe tubulointerstitial involvement and anemia has been demonstrated in patients with IgA nephropathy [18], similar associations have not been consistently confirmed across other PGN subtypes [19]. The present study extends the available evidence by evaluating this relationship in the largest multicenter PGN cohort to date, encompassing diverse histopathological diagnoses and a broad spectrum of renal function. The nationwide registry design and substantial sample size enhance the generalizability and robustness of our findings within PGN populations. Although experimental studies demonstrate that renal erythropoietin-producing interstitial cells undergo phenotypic transformation in fibrogenic microenvironments, leading to reduced EPO production, our findings suggest that in PGN this biological process may not translate into an independent clinical effect once overall renal function is accounted for [23]. This observation suggests that the apparent association between fibrosis and anemia may largely reflect its correlation with reduced renal function, rather than indicating an independent pathological mechanism; however, this interpretation should be considered associative rather than causal given the cross-sectional design. The nationwide multicenter design and large sample size enhance the external validity of the findings across diverse PGN populations.

Renal function remained the dominant determinant of anemia in our cohort, and the probability of anemia increased progressively as eGFR declined. This finding aligns with the well-established pathophysiological framework of chronic kidney disease-related anemia, in which reduced nephron mass and impaired erythropoietin production represent central mechanisms. Contemporary clinical guidance consistently emphasizes that anemia prevalence increases across CKD stages in parallel with declining GFR [22]. Large observational studies have reported that 21–62% of non-dialysis-dependent CKD patients exhibit anemia, with a stepwise rise in prevalence as kidney function deteriorates [1,2,3]. Notably, in a multicenter Chinese cohort of 2420 non-dialysis CKD patients―of whom approximately 60% had chronic glomerulonephritis―the prevalence of anemia increased from 22.4% in CKD stage 1 to 51.9% in stage 3 and 87.7% in stage 5 [6]. These data underscore that, irrespective of underlying etiology, CKD stage remains the principal determinant of anemia burden. Mechanistically, declining GFR reflects both quantitative nephron loss and functional impairment of renal erythropoietin-producing cells, leading to relative erythropoietin deficiency, impaired iron utilization, chronic inflammation, and altered hypoxia signaling pathways. Thus, even in diseases primarily characterized by glomerular pathology, the transition from structural injury to functional renal impairment appears to be the key mediator linking kidney disease to anemia.

In our PGN cohort, eGFR retained an independent association with anemia in multivariable models, whereas interstitial fibrosis did not. This pattern supports the interpretation that the apparent fibrosis–anemia relationship observed in unadjusted analyses is largely mediated through its association with reduced renal function rather than representing an autonomous pathological pathway. In other words, in PGN patients, functional renal reserve appears to be a stronger clinical determinant of anemia than histologic fibrosis grade alone. Importantly, we observed a statistically significant IF grade × eGFR interaction, visualized by model-based predicted probabilities. This finding indicates that the association between fibrosis grade and anemia varies across levels of renal function, consistent with effect modification in observed associations rather than a causal interaction. A plausible interpretation is that the clinical impact of structural interstitial injury on erythropoiesis becomes more discernible in the setting of reduced functional reserve, whereas at higher eGFR levels, compensatory mechanisms may predominate. Therefore, the observed interaction should be interpreted as a statistical phenomenon within the modeling framework, rather than as evidence of a direct biological interaction.

Female sex was independently associated with anemia in our multivariable models. This finding may partly reflect known sex-related differences in hemoglobin distribution and iron balance, as well as the sex-specific hemoglobin thresholds used to define anemia. Residual confounding by unmeasured iron indices and inflammatory markers is also possible, as these variables were not available in the registry. In this context, the persistent association of female sex with anemia supports the need for comprehensive anemia phenotyping (including iron indices and inflammation) when interpreting biopsy-based correlates of anemia.

We also observed higher odds of anemia among patients with membranoproliferative glomerulonephritis (MPGN) compared with FSGS, including within the preserved-eGFR subgroup model. MPGN comprises biologically heterogeneous entities and is often associated with systemic inflammatory or complement-mediated activity, which could contribute to anemia through inflammatory pathways and altered iron handling. Because CRP, ferritin/transferrin saturation, erythropoietin levels, and anemia treatments were not captured, we cannot determine whether the MPGN association reflects inflammation-related anemia, iron-restricted erythropoiesis, or other unmeasured mechanisms. Complement-mediated injury and chronic immune activation may further impair erythropoiesis through inflammatory iron sequestration pathways. Future studies incorporating standardized iron indices and inflammatory profiling are needed to clarify anemia phenotypes across PGN subtypes.

This registry analysis is limited by its cross-sectional design, lack of iron indices (ferritin, transferrin saturation), inflammatory markers, erythropoietin levels, and anemia treatments (iron/ESA), all of which influence hemoglobin and may confound associations. Because exposure (fibrosis severity) and outcome (hemoglobin/anemia) were measured at the same time point (biopsy), temporal direction cannot be established and reverse causality cannot be excluded. Single time-point laboratory measurements may not reflect longitudinal anemia status or treatment effects, and prospective follow-up analyses are needed. We did not account for potential center-level clustering effects, which may influence practice patterns and laboratory variability. Another limitation is the absence of albuminuria measurements. Although total proteinuria was used as a pragmatic and widely available measure across centers, albuminuria may have provided more specific information regarding glomerular injury and could have refined the interpretation of proteinuria-related associations. Histopathology was assessed locally at multiple centers without centralized review, introducing potential inter-observer variability that could attenuate true associations. Nonetheless, the nationwide scope and biopsy-confirmed diagnoses provide strong external validity for PGN populations and support the clinical interpretation that anemia risk is primarily driven by renal functional status, with fibrosis severity offering limited incremental information except through its interaction with eGFR.

## 5. Conclusions

In this large nationwide cohort of biopsy-proven primary glomerulonephritis, interstitial fibrosis severity was not independently associated with anemia after multivariable adjustment. Renal function was a major determinant of anemia, and the fibrosis–anemia relationship varied across eGFR levels as indicated by a significant IF×eGFR interaction. These findings suggest that, in PGN, anemia is primarily driven by functional renal impairment, while histological fibrosis alone provides limited incremental information.

## Figures and Tables

**Table 1:** Baseline clinical, laboratory, and histopathological characteristics of patients with primary glomerulonephritis, stratified by anemia status.

Variable	All Patients (*n* = 2794)	Anemic (*n* = 960)	Non-Anemic (*n* = 1834)	*p*-Value
Clinical characteristics				
Age (years)	40.0 (29.9–51.5)	42.1 (30.6–53.8)	38.8 (29.5–50.1)	<0.001
Male sex, *n* (%)	1591 (56.9%)	507 (52.8%)	1084 (59.1%)	0.004
Diabetes Mellitus, *n* (%)	260 (9.5%)	101 (10.8%)	159 (8.9%)	0.239
Hypertension, *n* (%)	924 (33.1%)	376 (39.9%)	548 (30.6%)	<0.001
Renin–angiotensin system inhibitors use, *n* (%)	1021 (36.5%)	370 (38.5%)	651 (35.5%)	0.112
Edema, *n* (%)	1323 (49.2%)	503 (54.3%)	820 (46.5%)	0.001
Primary Kidney Disease, *n* (%) Membranous nephropathy IgA nephropathy FSGS MPGN Mesengioproliferative GN Others	854 (30.6%) 856 (30.6%) 733 (26.2%) 253 (9.1%) 82 (2.1%) 16 (0.6%)	283 (29.5%) ^a^ 295 (30.7%) ^a^ 222 (23.1%) ^a^ 137 (14.3%) ^a^ 16 (1.7%) ^a^ 7 (0.7%) ^a^	571 (31.1%) ^a^ 561 (30.6%) ^a^ 511 (27.9%) ^b^ 116 (6.3%) ^b^ 66 (3.6%) ^b^ 9 (0.5%) ^a^	<0.001
CKD Stage, *n* (%) 1 2 3 4 5	1302 (47.1%) 632 (22.9%) 588 (21.3%) 176 (6.4%) 68 (2.5%)	305 (32.1%) ^a^ 180 (18.9%) ^a^ 279 (29.4%) ^a^ 127 (13.4%) ^a^ 59 (6.2%) ^a^	997 (54.9%) ^b^ 452 (24.9%) ^b^ 309 (17.0%) ^b^ 49 (2.7%) ^b^ 9 (0.5%) ^b^	<0.001
Body Mass Index (kg/m^2^)	26.6 (23.7–29.7)	25.9 (23.1–29.1)	27.0 (24.2–30.0)	<0.001
Laboratory characteristics				
BUN (mg/dL)	17 (12–25)	22 (14–35)	15 (11–21)	<0.001
Creatinine (mg/dL)	1.0 (0.7–1.5)	1.2 (0.8–2.0)	0.9 (0.7–1.3)	<0.001
eGFR (mL/min/1.73 m^2^)	85.6 (52.1–113.1)	60.5 (34.2–102.4)	94.8 (66.3–116.1)	<0.001
Proteinuria (mg/day)	3423 (1544–6400)	3662 (1700–6600)	3299 (1486–6257)	0.078
Albumin (g/dL)	3.5 (2.6–4.0)	3.2 (2.4–3.8)	3.6 (2.7–4.1)	<0.001
Uric acid (mg/dL)	6.1 (4.9–7.4)	6.2 (5.0–7.7)	6.1 (4.9–7.3)	0.051
Glucose (mg/dL)	91 (84–102)	91 (83–103)	91 (84–101)	0.796
HDL (mg/dL)	46 (38–58)	45 (37.8–59)	47 (38–58)	0.109
LDL (mg/dL)	144 (110–192)	142 (106–184)	145 (112–197)	0.018
Calcium (mg/dL)	9.0 (8.4–9.4)	8.7 (8.1–9.2)	9.1 (8.6–9.5)	<0.001
Alanine aminotransferase (U/L)	17 (13–24)	15 (12–21)	18 (14–25)	<0.001
Hematuria, *n* (%)	1329 (49.6%)	525 (56.3%)	804 (46.0%)	<0.001
Leukocyturia, *n* (%)	472 (17.7%)	212 (22.8%)	260 (15.0%)	<0.001
Histopathological findings				
Total glomeruli (*n*)	15 (10–23)	15 (10–23)	15 (10–21)	0.469
Globally sclerotic glomeruli (*n*)	1 (0–4)	2 (0–4)	1 (0–3)	0.002
Segmental sclerosis (*n*)	0 (0–2)	0 (0–2)	0 (0–2)	0.575
Interstitial fibrosis stage, *n* (%) Normal Mild Moderate Severe	1149 (41.1%) 1152 (41.3%) 394 (14.1%) 99 (3.5%)	355 (37.0%) ^a^ 380 (39.6%) ^a^ 177 (18.4%) ^a^ 48 (5.0%) ^a^	794 (43.3%) ^b^ 772 (42.1%) ^a^ 217 (11.8%) ^b^ 51 (2.8%) ^b^	<0.001
Tubular Atrophy stage, *n* (%) Normal Mild Moderate Severe	1078 (39.0%) 1256 (45.5%) 325 (11.8%) 104 (3.8%)	325 (34.4%) ^a^ 419 (44.3%) ^a^ 158 (16.7%) ^a^ 44 (4.7%) ^a^	753 (41.4%) ^b^ 837 (46.4%) ^a^ 167 (9.2%) ^b^ 60 (3.3%) ^a^	<0.001

Continuous variables are presented as median (interquartile range). Categorical variables are expressed as *n* (%). Group comparisons were performed using the Mann–Whitney U test for continuous variables and the chi-square test (or Fisher’s exact test when appropriate) for categorical variables. Superscript letters indicate significant pairwise differences in column proportions after Bonferroni correction.

**Table 2:** Multivariable logistic regression analysis of factors associated with anemia.

Predictor	β (Log-Odds)	Adjusted OR	95% CI	*p*
Mild IF vs. normal	−0.090	0.91	0.74–1.12	0.39
Moderate IF vs. normal	−0.038	0.96	0.72–1.28	0.79
Severe IF vs. normal	0.144	1.16	0.71–1.90	0.57
Female vs. male	0.506	1.66	1.39–1.98	<0.001
Age (per year)	−0.004	0.996	0.988–1.004	0.34
eGFR (per 1 mL/min/1.73m^2^)	−0.0211	0.979	0.975–0.983	<0.001
ln(Proteinuria +1)	0.057	1.06	0.97–1.16	0.18
Hypertension	0.087	1.09	0.89–1.33	0.41
Diabetes	0.142	1.15	0.85–1.57	0.36
IgA vs. FSGS	0.198	1.22	0.96–1.55	0.10
Membranous vs. FSGS	0.298	1.35	1.04–1.75	0.028
MPGN vs. FSGS	0.880	2.41	1.71–3.40	<0.001
Mesangioproliferative vs. FSGS	−0.330	0.72	0.37–1.41	0.34
Other vs. FSGS	0.052	1.05	0.36–3.08	0.93

Complete-case sample size: 2486 patients. Model AUC = 0.705. A test for linear trend across IF categories was performed by modeling IF severity as an ordinal variable in the regression model (*p*-trend = 0.72). Proteinuria was log-transformed as ln(mg/day + 1).

**Table 3:** Multivariable logistic regression model including interstitial fibrosis as a binary variable.

Predictor	β	Adjusted OR	95% CI	*p*
IF present vs. absent	−0.059	0.94	0.77–1.14	0.52
Female	0.501	1.65	1.38–1.98	<0.001
Age	−0.004	0.996	0.988–1.004	0.33
eGFR	−0.0211	0.979	0.975–0.983	<0.001
ln(Proteinuria +1)	0.051	1.05	0.97–1.15	0.21
Hypertension	0.093	1.10	0.89–1.34	0.39
Diabetes	0.146	1.16	0.85–1.58	0.34
IgA vs. FSGS	0.189	1.21	0.95–1.54	0.12
Membranous vs. FSGS	0.291	1.34	1.03–1.74	0.032
MPGN vs. FSGS	0.871	2.39	1.70–3.37	<0.001
Mesangioproliferative vs. FSGS	−0.326	0.72	0.37–1.40	0.35
Other vs. FSGS	0.050	1.05	0.36–3.08	0.93

Complete-case sample size: 2486 patients. Model AUC = 0.704. Proteinuria was log-transformed as ln (mg/day + 1).

**Table 4:** Complete-case multivariable logistic regression analysis of interstitial fibrosis severity and anemia in patients with preserved renal function (eGFR ≥ 60 mL/min/1.73 m^2^).

Variable	β (Log-Odds)	Adjusted OR (95% CI)	*p*
Mild IF vs. normal	−0.040	0.96 (0.76–1.22)	0.744
Moderate IF vs. normal	0.117	1.12 (0.74–1.72)	0.589
Severe IF vs. normal	−0.802	0.45 (0.15–1.31)	0.144
Female vs. male	0.384	1.47 (1.17–1.84)	<0.001
Age (per year)	0.004	1.00 (1.00–1.01)	0.348
ln(Proteinuria mg/day + 1)	0.029	1.03 (0.92–1.15)	0.596
Hypertension (yes vs. no)	−0.043	0.96 (0.73–1.26)	0.759
Diabetes (yes vs. no)	0.330	1.39 (0.95–2.05)	0.093
IgA nephropathy vs. FSGS	0.219	1.24 (0.89–1.73)	0.193
Membranous nephropathy vs. FSGS	0.375	1.46 (1.07–1.97)	0.016
MPGN vs. FSGS	0.927	2.53 (1.65–3.86)	<0.001
Mesangioproliferative GN vs. FSGS	−0.214	0.81 (0.38–1.73)	0.584
Other diagnosis vs. FSGS	0.544	1.72 (0.42–7.13)	0.453

Complete-case sample size: 1722. Model AUC = 0.604 eGFR was not included in this model. Proteinuria was log-transformed as ln(mg/day + 1).

## Data Availability

We created a database for primary glomerular diseases with the name of ‘Turkish Society of Nephrology Glomerular Diseases Working Group (TSNGOLD) in 04.04.2008. The data can be found at the following address: http://pgh.tsn.org.tr/login.php. Data are not publicly available due to legal and ethical reasons. Deidentified data are available upon reasonable request from the corresponding author.

## Abbreviations

The following abbreviations are used in this manuscript:

ALT	Alanine Aminotransferase
AUC	Area Under the Receiver Operating Characteristic Curve
BMI	Body Mass Index
BUN	Blood Urea Nitrogen
CI	Confidence Interval
CKD	Chronic Kidney Disease
CKD-EPI	Chronic Kidney Disease Epidemiology Collaboration
EPO	Erythropoietin
FSGS	Focal Segmental Glomerulosclerosis
GFR	Glomerular Filtration Rate
HDL	High-Density Lipoprotein
IF	Interstitial Fibrosis
IFTA	Interstitial Fibrosis and Tubular Atrophy
IgA	Immunoglobulin A
IQR	Interquartile Range
KDIGO	Kidney Disease: Improving Global Outcomes
LDL	Low-Density Lipoprotein
MPGN	Membranoproliferative Glomerulonephritis
OR	Odds Ratio
PGN	Primary Glomerulonephritis
REP	Renal Erythropoietin-Producing Cells
TA	Tubular Atrophy
TSN-GOLD	Turkish Society of Nephrology Glomerular Diseases Working Group Registry
VIF	Variance Inflation Factor
WHO	World Health Organization
